# Effect of electrical stimulation amplitude on dynamic behavior of mice during evacuation

**DOI:** 10.1016/j.heliyon.2023.e12930

**Published:** 2023-01-12

**Authors:** Duyen Thi Hai Nguyen, Hyeryon Seo, Junyoung Park

**Affiliations:** a Department of Mechanical Design Engineering, Kumoh National Institute of Technology, 61, Daehak-Ro, Gumi, Gyeungbuk 39177, Republic of Korea; b Department of Aeronautic, Mechanical and Electrical Convergence Engineering, Kumoh National Institute of Technology, 61, Daehak-Ro, Gumi, Gyeungbuk 39177, Republic of Korea

**Keywords:** Mice experiments, Evacuation, Desired velocity, Electric shock

## Abstract

Profound examination of the dynamic behavior of pedestrians during evacuation can significantly reduce the number of associated accidents. Conducting experiments on animals can help obtain deep insight into the dynamic behavior of pedestrians. Previous experiments using insects, such as ants and woodlice, showed large differences between the dynamic behaviors of insects and humans. However, systematic studies on the behavioral characteristics (e.g., velocity) of mice under electrical stimulation conditions have not been reported. Therefore, this study was conducted to investigate changes in the dynamic behavior of mice during evacuation caused by electric shock. Electrical stimulation was supplied through their feet during evacuation. The average velocity, desired velocity (maximum instantaneous velocity), average velocity in the congestion zone, and escape time were measured and analyzed. According to the results, the desired velocity and escape time increased in proportion to the amplitude of the electrical stimulation; however, the average velocity decreased. Consequently, the level of emergency of mice is affected by both the amplitude of electrical stimulation and the number density in congestion area as in human experiments.

## 1 Introduction

Numerous festivals and large events with thousands of participants are organized on a daily basis. The higher the number of people attending the event, the higher the risk of accidents. A recent example is the Jewish holiday of Lag BaOmer on April 30, 2021, in Mount Meron, Israel, with an estimated 100,000 attendees. A deadly stampede occurred [1] when people tripped and slipped; consequently, 45 people were killed and approximately 150 individuals were injured. On June 26, 2019, 16 people were killed and 101 individuals were injured in Antananarivo, Madagascar, because they joined the Madagascar city stadium before an Independence Day concert [2]. On October 3, 2005, at the Sangju Bicycle Festival in Sangju City, Gyeongbuk Province, South Korea, 11 people died and approximately 100 individuals were injured when an excessive number of spectators flocked to the stadium to attend concerts held during the festival [3]. These accidents are often caused by an incidental event that results in chaos in the crowd. In such cases, the individuals constituting the crowd want to exit at the highest possible speed [4]. Therefore, these accidents may be prevented by exploring the dynamic behavior of people when they evacuate in an emergency and the influence of external factors on the flow of people attempting to escape.

Human behavior during emergencies can affect the evacuation efficiency. In addition, the level of emergency influences human behavior. Therefore, the study of pedestrian flows during emergencies is of significant importance for crowd management. In evacuation scenarios, an emergency evacuation may be triggered by an incident that makes people feel extremely anxious, where individuals may push others to guarantee their own safety. However, reproducing the real evacuation of people for research purposes is challenging, because creating situations of real danger for humans to study their behavior during evacuation is impractical. Most experiments on the characteristics of human flow [5,6] have been performed with non-urgent crowds to guarantee the safety of participants. Some other experiments used hypothetical emergency conditions for humans. Emergency environments are created by instructing participants, using stressful auditory and lighting effects, or offering motivating rewards [[7], [8], [9], [10], [11]]. However, the extent of similarity between hypothetical and actual emergencies remains controversial. Indeed, reward- or guide-based experiments are different from the escaping pressure to save one's life in a real-danger condition. Therefore, simulations using computer models [4,[12], [13], [14], [15], [16]] and experimental studies on animals [17–24] have been conducted to obtain more realistic results. These studies have elucidated the dynamic behavior of participants attempting to escape an emergency and the degree of influence of factors leading to emergency conditions. Evacuation experiments have been performed with animals or insects, such as sheep, rodents, ants, and woodlice. In these experiments, the level of emergency was controlled using temperature, water, light, electric shock, or the smell of food. Zuriguel et al. conducted an experiment with sheep herds, in which evacuation (or congestion) was reproduced by enticing them to urgently move to obtain food [24]. Wang et al. tested the escape behavior of ants from a single room with a single exit [23]. In another study, Boari et al. tested the escape behavior of ants under an emergency situation caused by temperature variations [17]. Sobhani et al. studied the evacuation behavior and effects of the number density of woodlice using light and heat to create an emergency situation because light and heat are dangerous for woodlice [22].

Rodents have long been used in evacuation experiments because their dynamic behavior is similar to that of humans [25]. Morris et al. and Saloma et al. used a water maze to induce emergencies in rodents [[19], [20], [21]]. Morris' experiment was performed to test the memory and learning abilities of rodents, whereas in Saloma's experiments, fear of water was employed as the emergency factor. The results of Saloma's investigation showed that mice could learn quickly. The trained mice exhibited a self-organizing queuing behavior and escaped through the exit in bursts of different sizes, which were governed by power-law distributions depending on the exit width. However, as the mice escape, the emergence induced by water may not be sufficiently large to observe the bottleneck because they move significantly slower in water than on land. Another experiment using rodents was performed by Ref. [18]; in which smoke from burning joss sticks was used to induce emergencies [18]. This experiment aimed to verify the “Faster-Is-Slower” (FIS) phenomenon, a well-known dynamic behavior in evacuation, reported by Helbing [4]. Another similar study [26] simulated evacuation using electrical stimulation, which can be considered an excellent approach for controlling the parameters leading to emergencies and reproducing the same stimulating environment for each experiment. Using fixed electrical stimulations, Oh's experiment induced an emergency situation to study the effect of various guide walls on evacuation, thereby confirming the FIS effect [13]. Moreover, discrete element method (DEM) simulations were performed to confirm the experimental results and demonstrate that DEM simulations can be used to analyze other experimental cases of mouse behavior under emergency conditions. In addition, by applying electrical shock in a mouse experiment, Kim examined the effects of entrance restriction on the evacuation velocity and total evacuation time in a small number of mice [27]. The results demonstrated that placing a restricted entrance could improve the evacuation efficiency.

Therefore, this study was performed to evaluate the effect of electrical stimulation amplitude on the movement behavior of mice during evacuation. Evacuation experiments with a large number of mice escaping from a room owing to an electric shock are presented. The remainder of this paper is organized as follows: The experimental procedure is described in Section 2. Through motion characteristics such as velocity and time, the results showing the behavior of mice are presented in Section 3. A thorough discussion is presented in Section 4. Finally, the conclusions are presented in Section 5.

## 2 Material and methods

### 2.1 Method selection

Many experimental studies in pedestrian evacuation fields have been performed in the past; however, there is a lack of research in this area under emergency conditions owing to concerns regarding the safety for participants. Mice were chosen for these experiments, because their brains contain the amygdala and hippocampus responsible for the fear state, similarly as in humans [25]. Therefore, mice are expected to respond similarly as humans to anxiety and fear. Moreover, mice also possess the ability to learn and remember, as humans do, rendering it easy to design evacuation experiments [19].

Although electric shocks are not normally experienced in actual emergencies, they are advantageous toward providing emergency stimulus in a laboratory, particularly because it is easily quantifiable. The electrical device used in this study can provide three controllable parameters: amplitude, duration, and frequency of event (accident).

### 2.2 Mouse model

For the experiments, 28 female and 28 male C57BL/6 N mice were procured and bred at the In Vivo Research Center, Ulsan National Institute of Science and Technology (UNIST), South Korea. Their age, width, length, height, and weight values were in the ranges of 9–11 months, 2.5–3 cm, 7.8–9.8 cm, 2.6–3.0 cm, and 25–35 g, respectively. Although the mice were trained to remember the locations of the narrow exit, a few mice were still running in the opposite direction of the narrow exit. Therefore, to obtain representative data for Group 1 (15 mice) and Group 2 (50 mice), we performed experiments with 16 mice and 56 mice. Correspondingly, data from one mouse and six mice with unusual movements were removed during analysis. The group of 16 mice comprised 8 female and 8 male mice.

### 2.3 Ethics declaration

Experiments were conducted by authors who have completed educational courses in research ethics. In addition, experiments were performed at UNIST following both the animal protection law and experimental protocol under the supervision of the Institutional Animal Care and Use Committee (IACUC), approved by IACUC (Authorization No. UNISTIACUC-16-21).

### 2.4 Experimental devices

**Fig. 1:**
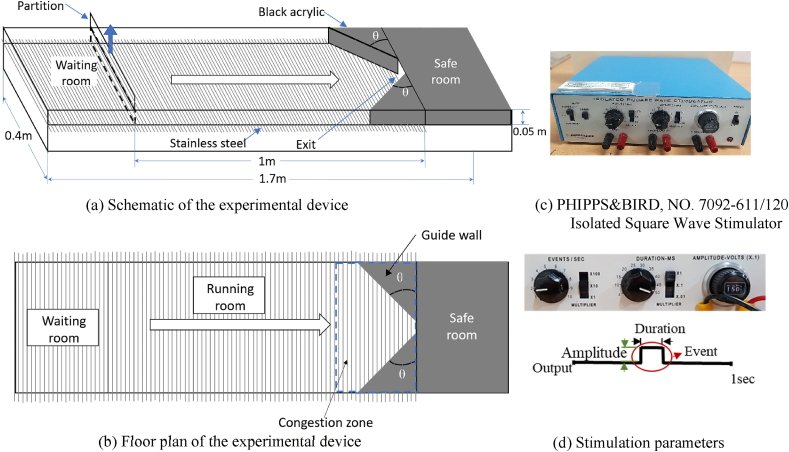
Schematics and photographs of experimental devices.

All experiments were performed using a device with waiting, running, and safety rooms, as shown in Fig. 1(a) and (b). The height of the experimental device was 0.05 m, which is sufficient for mice to move easily, while ensuring to eliminate their jumping behavior. This experimental device primarily comprised a transparent acrylic wall and stainless-steel rods used to transmit electricity to the mice through their feet. Electrical stimulation was applied by connecting the waiting and running rooms to the electrical equipment to induce emergencies during evacuation. The waiting and running rooms were separated by a partition. The guide wall and safety room were darkened considering that the mice preferred dark spaces. The narrow exit was located at the center between the running and safety rooms. It had a width of 5 cm, which is barely wide for two mice to escape simultaneously. A guide wall at an exit angle of 45° was installed at the exit to funnel the mice into a safe room. The device used to provide the electrical stimulus was a Phipps & Bird Isolated Square Wave Stimulator 7092-61, as shown in Fig. 1(c). It provides three controllable parameters: amplitude (mV), duration (ms), and event (s^−1^), as presented in Fig. 1(d). An FDR-AX700 (Sony) camcorder was attached to the experimental device to record the entire experimental procedure.

### 2.5 Experiment procedure

The dynamic behavior of mice shows a collective movement behavior similar to that of humans [25]; however, it cannot be controlled with language communication, as in human crowds. Therefore, they were trained to recognize exit locations and easily move up to the exit point. During the experiment, the mice were held in a waiting room and subjected to an electric shock. A few seconds after the shock, the mice panicked and were stamped at the exit. At this point, the partition was opened, and recording was initiated. After the final mouse escaped into the safe room through the narrow exit, recording was stopped.

The increase in electric shock amplitude depended on the tolerance of the mice. Preliminary tests showed that the effect of electric shocks with an amplitude of less than 40 V on the mouse behavior was negligible. Therefore, in this study, the electric shock amplitude was increased from 40 to 55 V in 5-V increments. The experiments were conducted thrice for each shock amplitude. The actual experiment was performed with two groups of mice under the same experimental conditions, that is, an exit angle of 45° and 200 “events” (or electric shocks) per second, each with a duration of 1 ms. To prevent exhaustion and stress, the mice were allowed to briefly rest with access to food and water after each “escape” run. During the evacuation, there were a very small number of rats riding on each other. However, the ride time was short, and it usually happened in the vicinity of the entrance, rather than at the exit when the partition was opened. Therefore, this phenomenon was not specifically considered in the analysis.

**Fig. 2:**
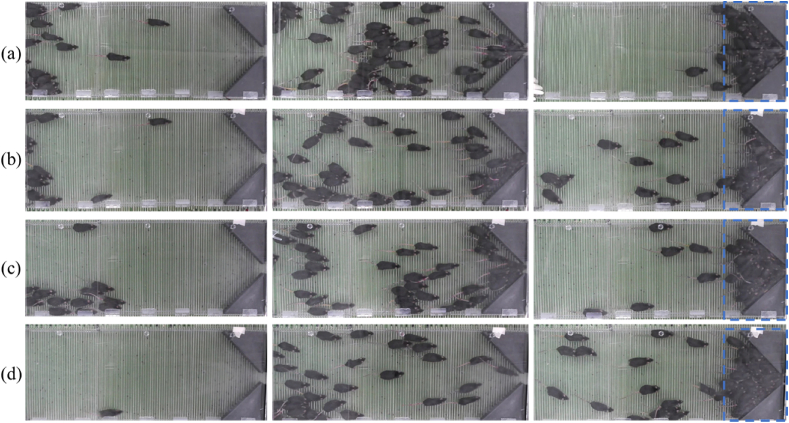
Snapshots of mice evacuation. The partition was opened after 1, 5, and 10 s from left to right, and the electric shock amplitude was (a) 40, (b) 45, (c) 50, and (d) 55 V.

The videos were recorded by the camcorder and transferred to a computer for analysis using an in-house code. The position of each mouse was extracted to the XY coordinates for each frame, allowing the analysis of the instantaneous velocity of each mouse. Fig. 1 shows the behavior of the mice in the cases where the partition was opened after 1, 5, and 10 s with an increasing amplitude of electric shocks, which increased from 40 V (Fig. 2(a)) to 55 V (Fig. 2(d)) in 5 V increments.

### 2.6 Data analysis

The videos were converted into image files and then analyzed using the MouseTracker program, an in-house code for obtaining the position of each mouse and analyzing data based on the following formulas and definitions.

The average velocity, $V_{average}$, of all mice in all cases with the same amplitude and number of mice can be expressed as:

(1) $$V_{average}=\frac{\sum_{j=1}^{j=C\;}\;\frac{\sum_{i=1}^{i=N}V_{mouse}}{N}}{C}\text{,}$$

where *C* is the number of cases per amplitude value (in this study, *C* is 3); *N* is the number of analyzed mice in that experiment; and $V_{mouse}$ is the average velocity of each mouse in the experiment, which is expressed as:

(2) $$V_{mouse}=\frac{\sum_{i=1}^{i=n}V}{n}\text{,}$$

where *n* denotes the number of measurements and V is the instantaneous velocity measured every 0.5 s (the frame rate to obtain the picture for calculating the position of each mouse). The average velocity of each mouse (V_*mouse*_) was calculated from the time it started in the running room until it passed the narrow exit.

The other parameters considered for the experiment were $t_{average}$ and $T_{average}$. Moreover, $t_{average}$ is the average value of the evacuation time for each mouse, which is expressed as follows:

(3) $$t_{average}=\frac{\sum_{j=1}^{j=C\;}\;\frac{\sum_{i=1}^{i=N}t_{mouse}}{N}}{C}\text{,}$$

where $t_{mouse}=t_{mouse,exit}-t_{mouse,start}$ is the evacuation time of each mouse.

The average of total evacuation time, $T_{average}$, can be calculated as follows:

(4) $$T_{average}=\frac{\sum_{i=1}^{i=C}T_{evacuation}}{C}\text{,}$$

where $T_{evacuation}=t_{last\;mouse,exit}-t_{first\;mouse,start}$ is the evacuation time of one experiment with *N* mice.

Additionally, congestion in front of the exit was considered, and parameters associated with the congestion zone were defined. The congested areas and congestion thresholds are dependent on time and other factors. There were no clear definitions for congestion thresholds. Therefore, we chose the value, 10 mice which occurs a small congestion, based on the observation during the experiments. The congestion zone was defined as a 0.4 m × 0.2 m rectangular area (dashed blue line shown at Fig. 2) in front of the exit door. The parameters associated with the congestion were calculated when there were at least 10 mice in the congestion zone. Moreover, $V_{mouse\_Cong}$ and the average velocity of the mice in the congestion zone, $V_{avr\_Cong}$, were considered and analyzed. These parameters were calculated similarly as those for determining the velocity, $V_{mouse}$, and average velocity, $V_{average}$; however, they are applied only within the congestion zone.

## 3 Results

### 3.1 Comparison with existing research

**Fig. 3:**
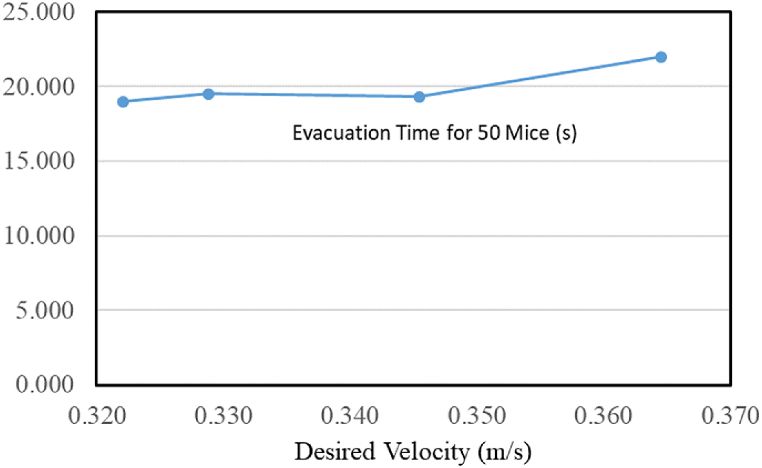
Evacuation time for a group of 50 mice.

Pedestrian evacuation studies are performed through various methodologies. In animal-based experimentations, different animals such as sheep, ants, rodents, and woodlice are used for experiments. Many methods have been applied to induce emergency conditions: smoke, electricity, heat, and lures with food. Comparing the results between experiments under such different conditions is limited to yielding only the trend. The use of electric shock to create an emergency condition by Ref. [13]; aimed to examine the effect of guide wall changes on the movement behavior of mice. Kim et al. [27] applied electric shock to investigate the effects of an obstacle placed before a narrow exit. These studies differ from our goals; therefore, the comparison will not give a good result. Fig. 3 shows the relationship between leaving time and desired velocity for 50 mice. The direct comparison between human behavior in simulations and mice behavior in the experiment cannot be established. However, an increasing trend in the evacuation time under the desired velocity increase could be observed. Helbing reported a similar trend based on the simulation of pedestrian evacuation [4].

### 3.2 Average and maximum instantaneous velocities

**Fig. 4:**
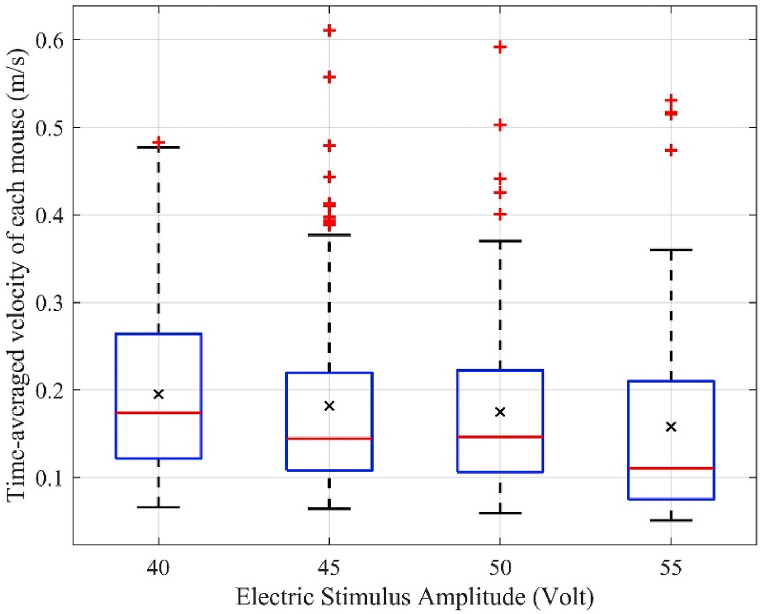
Distribution of average velocities in a group of 50 mice at different electric stimulus amplitudes.

Fig. 4 shows the box plot for the time-averaged velocity of each mouse, $V_{mouse}$ was calculated using Equation (2), when the amplitude of the electric stimulus changes in the range of 40–55 V in the case of experiment analysis with 50 mice. The black cross “**×**” and red plus “**+**” marks represent the average velocity of the mice, $V_{average}$, and the outlier points of the velocity of the mice, respectively. The speed of each mouse was expected to be proportional to the strength of the electrical stimulation. However, a marginally decreasing tendency in the average velocity of the mice, $V_{average}$, with increasing amplitude of the electric stimulus was observed, which is ascribed to the level of emergency in the mice. The appearance of several red plus marks indicates that at high amplitudes of electric stimulus, few mice tended to run extremely fast. However, because the majority of mice moved at lower velocities, the overall trend of the average velocity was downward. The velocity distribution range of the mice (i.e., the length of the vertical dashed line) is the largest at 40 V and shortens as the amplitude of the electric stimulus increases.

**Fig. 5:**
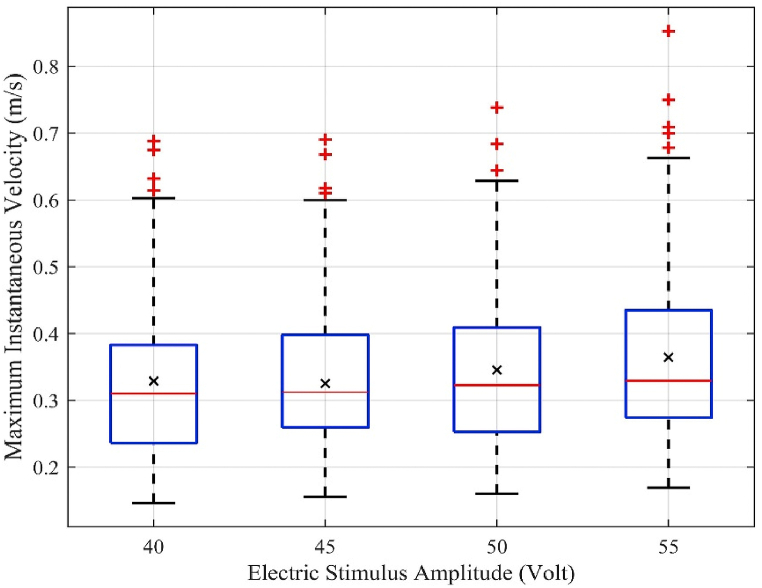
Distribution of the maximum instantaneous velocity in a group of 50 mice at different electric stimulus amplitudes.

During escape, the instantaneous velocities of mice were not always constant. The time-averaged velocity, $V_{mouse}$, of each mouse was calculated by averaging the instantaneous velocities. The desired velocity of mice, which is the running velocity in a certain situation without any interference from other mice, is a significant measure of the degree of emergency. The more emergent the mouse, the greater the desired velocity of the mouse. The maximum instantaneous velocity of mice was close to the desired velocity. Therefore, the maximum instantaneous velocity of mice during an emergency is a reasonable indication of the degree of emergency. Fig. 5 shows the box plot of the maximum instantaneous velocity of the mice in the experiment analysis case with 50 mice. As the amplitude of the electric stimulus increases, both the mean value “×” and distribution of the maximum instantaneous velocity (box and dashed line) exhibit a marginally increasing trend. However, the distribution range (i.e., the length of the dashed line) did not seem to change significantly. Thus, the desired velocity and level of emergence in all mice increased with increasing electric stimulus amplitude.

### 3.3 Observation of the congestion zone

**Fig. 6:**
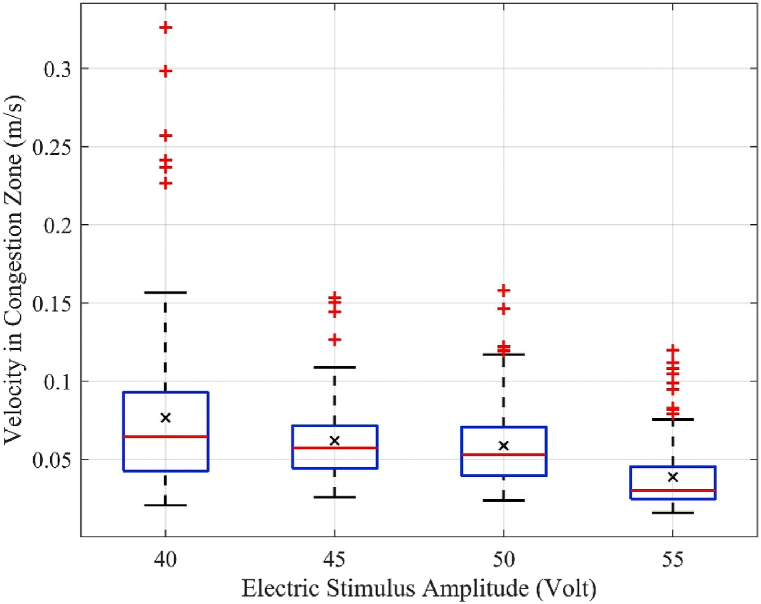
Distribution of the average velocities in a group of 50 mice in the congestion zone.

The average velocity of the mice in the area near the exit, $V_{avr\_Cong}$, is a key parameter, because this area is highly prone to congestion. The lower the speed of movement in this area, the more impatient the crowd, which may lead to jostling and accidents in human congestion conditions. In this experiment, the congestion zone is defined as a 0.4 m × 0.2 m rectangular area in front of the exit door, based on the observation. Fig. 6 shows the box plot of the velocity of the mice measured solely in the congestion zone, $V_{mouse\_Cong}$ in the experiment analysis case with 50 mice. It can be observed that the average velocity in the congestion zone, $V_{avr\_Cong}$, depicted by “×” decreases as the electric stimulus amplitude increases. In addition, the distribution range of velocities becomes significantly narrower with increasing amplitude. At an electric shock amplitude of 55 V, majority of mice in the congestion zone had a relatively low velocity (<0.05 m/s).

### 3.4 Evacuation time

**Fig. 7:**
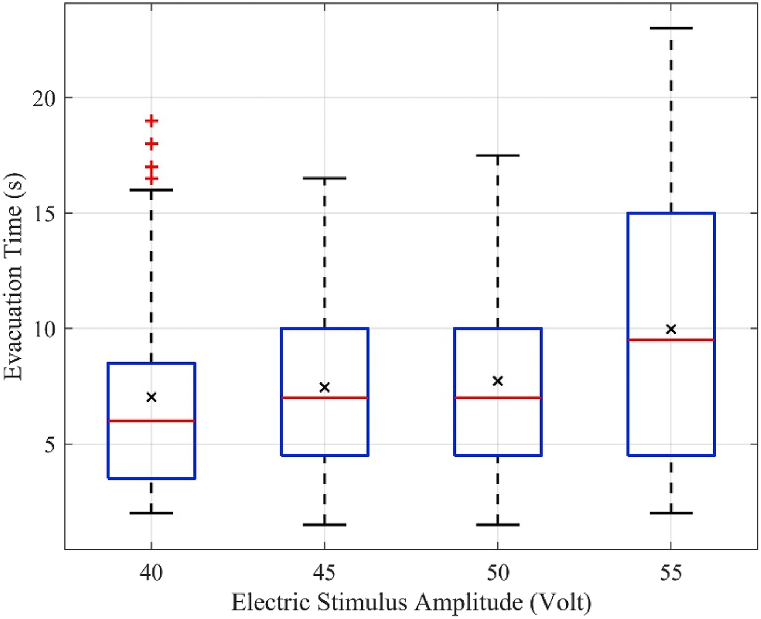
Distribution of the evacuation time in a group of 50 mice at different electric stimulus amplitudes.

Fig. 7 shows a box plot of the evacuation time of each mouse, $t_{mouse}$ in the experiment analysis case with 50 mice. When the electric shock amplitude increases, the average value of evacuation time, $t_{average}$ was calculated using Eq. (3), depicted by “×”, increases. In addition, the distribution range of the evacuation time becomes wider. Interestingly, the minimum values of the evacuation time for all four electric shock amplitudes were comparable. However, the maximum value increased with increasing electric shock amplitude. The minimum evacuation time was the evacuation time of the fastest mouse, whereas the maximum value was that of the slowest mouse. Therefore, this evacuation time change indicates that the escape time of the mice that escaped quickly remained the same and the number of mice that escaped later increased.

### 3.5 Comparison of the results of the two groups of mice

**Fig. 8:**
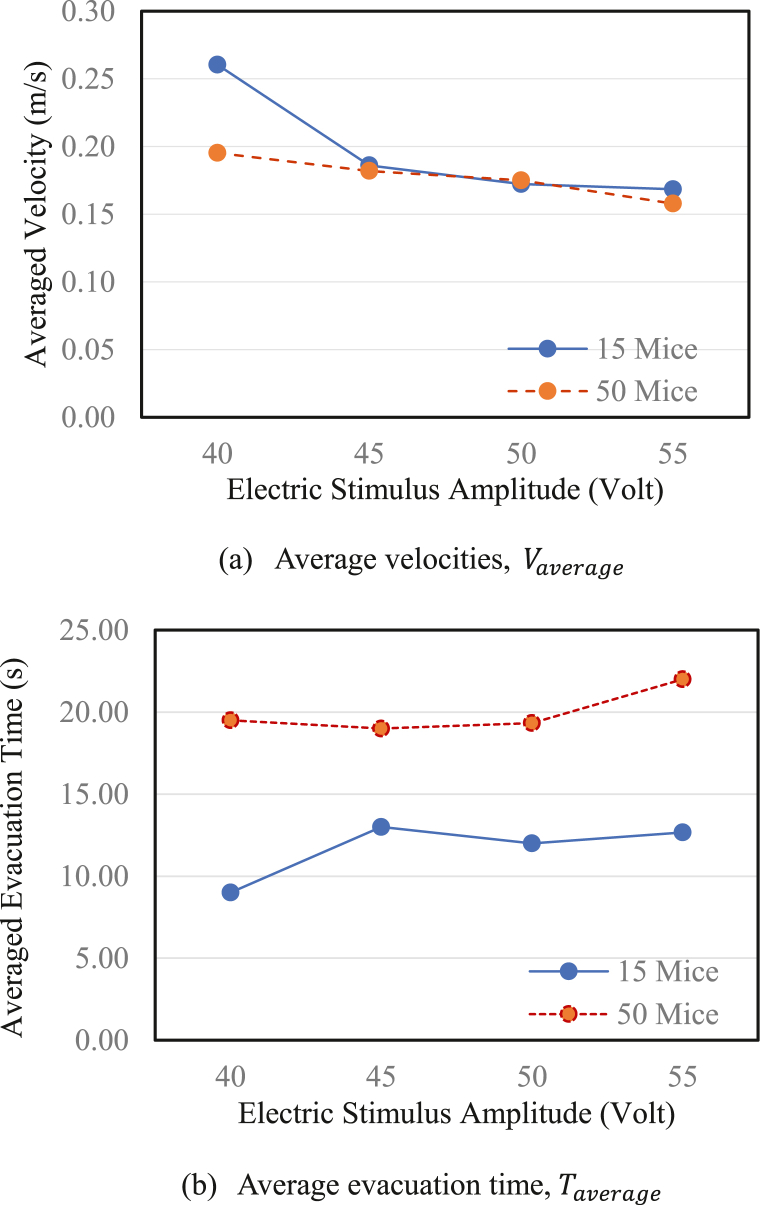
Comparisons of the average (a) evacuation time and (b) velocity between the groups with 50 and 15 mice.

In this study, experiments were conducted on Groups 1 and 2 with 15 and 50 mice, respectively. Fig. 8 shows the average velocity, $V_{average}$, and the average of total evacuation time, $T_{average}$, of mice in all cases, which were calculated using Eqs. (1) and (4). In general, both the average velocity and average evacuation time exhibited the same tendency in the two groups. Moreover, whereas the average velocity exhibited a decreasing tendency, the total evacuation time showed an increasing tendency. This was expected, because the velocity and evacuation travel time were inversely proportional. Fig. 8(b) only shows the meaning of tendency, because evidently, the larger the number of mice, the greater evacuation time. However, Fig. 8(a) shows that, in addition to exhibiting the same decreasing trend, when the amplitude is greater than 45 V, the average velocity appears to be approximately equal between Groups 1 and 2. In Group 2, when the electric shock amplitude increases from 40 to 45 V, the total evacuation time decreases marginally. The same phenomenon is observed in Group 1 when the electric shock amplitude increases from 45 to 50 V “Faster-Is-Slower” (FIS) is a well-known phenomenon in pedestrian evacuation. It means the faster you go, the later you get. This phenomenon partially appears in Groups 1 and 2 when the amplitude increases from 45 to 50 V and from 40 to 50 V, respectively.

Moreover, the mice exhibited a temporary freeze in emergencies. This behavior is a response to an emergency condition caused by electrical stimulation supplied through the feet [28]. The observation of the recorded videos from the experiment clearly showed that the mice stopped moving several times during the experiment; however, this “frozen” state must be quantified using the criterion of instantaneous velocity to compare the effects of various emergency conditions. In this study, freezing was considered to constitute stopping or slow movements of mice corresponding to an instantaneous velocity of less than 0.01 m/s, as presented in Table 1. In both groups, the number of times the mice froze gradually increased with increasing electric shock amplitude, indicating that the level of emergency gradually increased. This value is extremely high at an electric shock amplitude of 55 V, indicating that the mice freeze more frequently at higher amplitudes of electric stimulus. The number of freezing instances, that is, the level of emergency, for Group 1 monotonically increased, whereas that for Group 2 was approximately linear when the amplitude increased from 40 to 50 V; however, it increased sharply at 55 V. Therefore, when the emergency is extremely high and the number of mice in evacuation is high, the mice panic and react with a higher freezing rate.

**Table 1:** Total number of times of mice freezing in the evacuation.

Electric shock amplitude	40 V	45 V	50 V	55 V
Group 1: 15 mice	11	19	30	33
Group 2: 50 mice	74	103	113	350

## 4 Discussion

The experiments were conducted to study the effect of the electric stimulus amplitude on the level of emergency during the evacuation of the mice. As listed in Table 1, an increasing amplitude increases the level of emergency in mice during their evacuation.

The average velocity, maximum instantaneous velocity, average velocity in the congestion zone, and escape time were analyzed and discussed. When the electric stimulus amplitude increased, the average velocity of the mice in the running room (Fig. 4) and average velocity in the congestion zone decreased (Fig. 6). However, the average maximum instantaneous velocity (Fig. 5) and average evacuation time increased (Fig. 7). The maximum instantaneous velocity is the desired velocity, and the average velocity is the actual velocity of the mice. The inverse proportion between the desired velocity and the average velocity indicates that the mice could not move at the desired velocity, similar to the dynamic behavior of humans in evacuation experiments.

The reaction intensity of mice increased with increasing electric shock amplitude. However, despite causing more fear and an increased desire to leave the room in the mice, higher amplitudes caused the mice to stop more often. The desire to exit the room quickly causes the desired velocity to increase; thus, the instantaneous velocity increases. However, the mice used in this experiment sometimes stopped responding to environmental conditions. The number of stops proportionally increased with an increase in the emergency level. Furthermore, stopping implies that the instantaneous velocity is approximately zero, and the mouse velocity (*V*_*mouse*_) is the average of instantaneous velocities; therefore, more stops lead to a lower average velocity.

However, although 15 or 50 mice received the same amount of electricity, the number of freezing in the 50 mice group was higher. According to the observations as well as the definition of the congestion zone and the congestion threshold, the group with 15 mice did not exhibit congestion during the evacuation. The number of freezing can be used as an indication of only the emergency level in this case. However, in the experiment with 50 mice, congestion occurred frequently, and the freezing increased sharply. This indicates that the level of emergency was increased by electricity, however, the level of emergency was also increased by the extent of congestion (i.e., the number of mice). In particular, for 55 V events, in the 50 mice group, the level of emergency significantly increased. Therefore, as the electrical stimulation amplitude increased, the level of emergency increased in high-density areas, reducing the speed in congestion areas, and increasing evacuation times. Thus, the competition and emergency levels of mice were affected, resulting in decreased average speeds and increased evacuation times.

## 5 Conclusions

In this study, we experimented with mice that escaped through narrow exits under emergency conditions. In addition, the effect of the electric shock amplitude was investigated. The results clearly demonstrated changes in the average velocity, desired velocity, average velocity in the congestion zone, and total evacuation time with varying amplitude values. The two groups of 50 and 15 mice exhibited the same tendency. Moreover, the FIS phenomenon was partially observed in both groups. In both groups, the number of times that the mice froze during evacuation increased with increasing electric shock amplitude. In addition, the extent of congestion increased the number of freezing events. In summary, the level of emergency was affected by both the amplitude of electrical stimulation and the number density in the congestion area. Moreover, the experimental results could provide a data set to simulate mouse movement by the social force model in future work.

## Author contribution statement

Duyen Thi Hai Nguyen: Conceived and designed the experiments; Performed the experiments; Analyzed and interpreted the data; Contributed reagents, materials, analysis tools or data; Wrote the paper.

Hyeryeon Seo: Performed the experiments; Analyzed and interpreted the data; Contributed reagents, materials, analysis tools or data.

Junyoung Park, PhD: Conceived and designed the experiments; Wrote the paper.

## Funding statement

This work was supported by Ministry of Science and ICT, South Korea [IITP-2022-2020-0-01612], National Research Foundation of Korea (NRF) [NRF-2018R1A2B 2004207].

## Data availability statement

Data will be made available on request.

## Declaration of interest's statement

The authors declare no conflict of interest.
